# Superb Microvascular Imaging Technology Can Improve the Diagnostic Efficiency of the BI-RADS System

**DOI:** 10.3389/fonc.2021.634752

**Published:** 2021-06-24

**Authors:** Siman Cai, Hongyan Wang, Xiaoyan Zhang, Li Zhang, Qingli Zhu, Qiang Sun, Jianchu Li, Yuxin Jiang

**Affiliations:** ^1^ Department of Medical Ultrasound, Peking Union Medical College Hospital, Chinese Academy of Medical Science and Peking Union Medical College, Beijing, China; ^2^ Department of Breast Surgery, Peking Union Medical College Hospital, Chinese Academy of Medical Science and Peking Union Medical College, Beijing, China

**Keywords:** super microvascular imaging, vascular index, vascular architecture, BI-RADS, breast examination

## Abstract

**Background:**

To explore whether superb microvascular imaging (SMI)SMI can improve the diagnostic efficiency by evaluating the vascular index (VI) and vascular architecture (VA) in breast lesions.

**Methods:**

This is a retrospective study of data collected prospectively for research use. Taking 225 consecutive cases of breast lesions from November 2016 to December 2017 as a training set, the VI values and VA types of benign and malignant lesions were calculated based on the pathological results. Taking 238 consecutive cases of breast lesions from January 2018 to October 2018 as the verification set, the diagnostic sensitivity, specificity, accuracy, positive predictive value (PPV) and negative predictive value (NPV) were calculated to compare the diagnostic efficacy.

**Results:**

The training set included 225 breast lesions and the validation set 238 breast lesions. The VI value in the malignant group (10.3 ± 8.0) was significantly higher than that in the benign group (4.3 ± 5.0)(P<0.001). A VI value of 4.05 was used as the diagnostic threshold for differentiating benign from malignant lesions, with a sensitivity of 80.5%, a specificity of 61.9%, an accuracy of 71.1%, a PPV of 62.9%, a NPV of 76.9%, and an area under the curve of 0.758 (0.696-0.819). There was a significant difference in the types of benign and malignant VA (P < 0.001), and the PPV of the root hair-like and crab claw-like VAs were 93.9% and 100.0%, respectively. The diagnostic sensitivity, specificity, accuracy, PPV, NPV and area under the AUC curve were 58.0%, 98.2%, 97.0%, 70.3% and 0.781, respectively (95%CI: 0.719-0.844). SMI combined with conventional ultrasound improved the diagnostic specificity (70.0% *vs.* 90.0%), accuracy (87.4% *vs.* 96.6%), and PPV (82.5% *vs.* 93.2%) without decreasing the diagnostic sensitivity (99.3%), yielded higher diagnostic performance with the area under the ROC curve was 0.941 (95%CI: 0904-0.979) compared with conventional US alone (P < 0.001).

**Conclusion:**

A VI value 4.05 is a cut-off value with good diagnostic efficacy. The residual root-like and crab claw-like VAs are the characteristic VAs of malignant lesions. Conventional ultrasound combined with the VI and VA can improve the diagnostic specificity, accuracy and PPV without reducing the diagnostic sensitivity.

## Introduction

Breast cancer is the most common malignant tumor in women and the leading cause of cancer-related deaths (1, 2). However, with the development of imaging diagnosis technology, earlier and smaller breast cancers can be diagnosed early through advanced imaging tools, and the five-year survival rate of breast cancer is improved (3). Breast Imaging Reporting and Data Systems (BI-RADS) of the American Society of Radiology is widely used in the evaluation of breast lesions and is the application guide of clinical routine breast ultrasound examinations. However, relevant studies have shown that BI-RADS has high sensitivity and low specificity for the diagnosis of benign and malignant breast lesions (4). The information at other levels provided by the new ultrasound technology is conducive to improving the accuracy, sensitivity, and specificity of ultrasound in the diagnosis of breast lesions, enhancing the confidence in the diagnosis of malignant lesions, and at the same time reducing the unnecessary biopsy and surgery of benign lesions (5, 6). Angiogenesis plays a crucial role in tumor genesis, growth, invasion and metastasis. Studies have shown that the density of microvessels in malignant breast lesions is significantly higher than that in benign lesions (7). Compared with benign lesions, malignant lesions have more chaotic and branching blood vessels (8). Therefore, the evaluation of blood flow abundance and architecture can improve the diagnostic efficacy of ultrasound in breast lesions. How to quickly, effectively, noninvasively, and comprehensively evaluate the blood vessels in breast lesions and provide microvessels information for the diagnosis is one of the urgent problems to be solved.

Superb microvascular imaging (SMI) is an innovative doppler ultrasound technology, through the analysis of the characteristics of motion artifacts, extract the clinically relevant information, using adaptive algorithm suppress clutter signal generated by tissue motion to reduce motion artifacts and display microvascular without injecting contrast agent (9, 10). Multiple studies have shown that in superficial tissues and organs, digestive system, musculoskeletal disorders, obstetrics and gynecology diseases, etc. SMI can be used to diagnose and evaluate tumors, inflammation, and injury to improve diagnostic efficiency (11–19). Smart 3D-SMI can reconstruct 3D images by scanning 2D images with linear array probes, realize 3D pattern visualization, and comprehensively observe the vascular architecture (VA) in detail. At the same time, the vascular index (VI) can be measured by delineating the edge of the lesion on the SMI image to quantitatively evaluate the abundance of blood flow, which represents the ratio of doppler signal pixel to the whole lesion pixel (20). At present, preliminary studies (21) have shown that SMI contributes to the diagnosis of benign and malignant breast lesions, but there is still a lack of systematic evaluation study on the diagnostic efficacy of SMI combined with conventional ultrasound for BI-RADS.

## Materials and Methods

### Patients and Lesions

**Figure 1:**
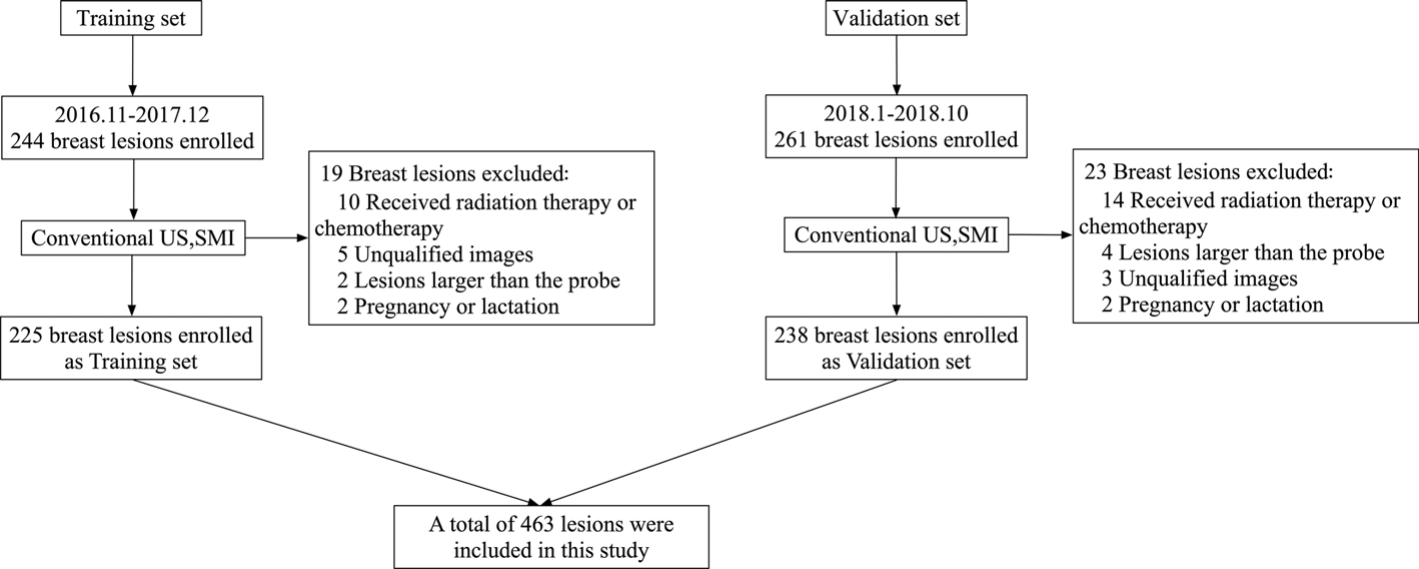
Flow chart of patients enrolment in the training set and verification set.

The Ethics Committee of Peking Union Medical College Hospital approved this prospective study. All patients were aware of the examination process and provided written informed consent. This is a retrospective study of data collected prospectively for research use. From January 2016 to November 2018, 504 consecutive patients were diagnosed by routine ultrasound and SMI diagnosis, and underwent biopsy or surgery were included in this study. An analysis of 244 breast lesions from 244 patients from November 2016 to December 2017 was performed as the training set. A total of 261 lesions from January 2018 to October 2018 were collected as the validation set. The study flow chart is shown in **Figure 1**.

The inclusion criteria were as follows: lesion size <6 cm (no more than the maximum scope of the probe display); no ipsilateral breast puncture, surgery or chemoradiotherapy, which can affect the blood supply of the lesion; patients who were not pregnant or in lactation, because the changes in hormone levels can affect the vascularization; and those with complete clinical information and written informed consent.

Training set: A total of 19 cases were excluded, 225 cases were included as the training set. A total of 112 lesions were malignant in 112 women (mean age 51.3 ± 12.4 years), and 113 lesions were benign in 113 women (mean age, 41.0 ± 11.0 years).

Validation set: A total of 23 lesions were excluded, 238 lesions from 238 patients were included as the validation set. A total of 138 lesions were malignant in 138 women (mean age 51.7 ± 12.8 years), and 100 lesions were benign in 100 women (mean age, 43.3 ± 12.3 years).

### US Examinations

Ultrasonographic examinations were performed using an Aplio 500 (L14-5, Aplio 500; Canon Medical Systems, Tokyo, Japan). All conventional two-dimensional ultrasound and Smart 3D-SMI architecture images were stored by a doctor with 15 years of experience in breast ultrasound examination. Vascular index examinations were independently performed by two ultrasound doctors (a doctor with 15 years of experience in breast ultrasound examination and a doctor with 3 years of experience in breast ultrasound examination), and the VI values were measured 3 times by the two doctors and averaged. The VA rating of the same lesion was independently evaluated by two doctors (a doctor with 4 years of breast ultrasound experience and a doctor with 3 years of breast ultrasound experience) who were blinded to the pathological results based on the images taken by the senior doctor (a doctor with 15 years of experience in breast ultrasound examination). The Smart 3D-SMI machine was set as follows: low speed of 1.0-2.0 cm/s, the frame rate of 25-30 f/s, pulse repeat frequency of 15.4-20.2 kHz, depth range adjusted to 2.5-4 cm according to the size of the lesion, and measured width of the linear array probe of 6 cm.

**Figure 2:**
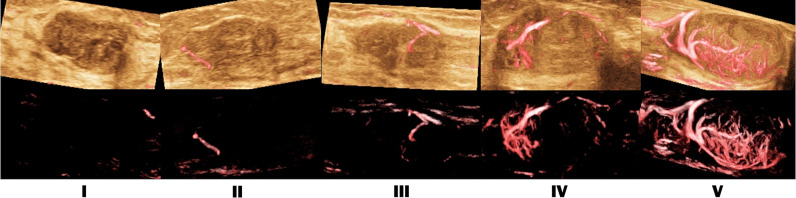
SMI vascular architecture (VA) types. I. no vascular pattern; II. dotted and linear pattern; III. tree-like pattern; IV. residual root-like pattern; V. crab claw-like pattern.

Doctors first scanned the breast with conventional ultrasound. Once suspicious lesions were found, classified according to the fifth edition of the “Breast Imaging Reporting and Data System” (BI-RADS) of the American College of Radiology (22). If the patient has multiple lesions, the most suspicious lesions were selected for the study. In Smart 3D-SMI mode, the lesions were detected by a uniformly moving probe, and the three-dimensional vascular image was reconstructed from a two-dimensional SMI image with a one-button action. Based on Smart 3D-SMI and other studies on the morphology and distribution characteristics of breast microvessels (9, 23), the VA in lesions was divided into the following 5 types (**Figure 2**): Type I, no vascular pattern, namely, lack of blood vessels in the lesion; Type II, dotted and linear pattern, single or a few dotted or linear areas with the absence of cross vessels observed in the lesion; Type III, tree-like pattern, with microvessels branching proportionally into the lesion; Type IV, residual root-like pattern, with mainly distorted and disordered arrangement of lesions with irregular blood vessels and less than two thick and distorted blood vessels around the lesions; Type V, crab claw-like pattern, characterized by radial vessels, with more branching vessels in the surrounding area. If the two doctors had inconsistent judgments, the senior doctor decided the final VA type. Smart 3D-SMI was used as a qualitative guide to identify the two-dimensional SMI plane with the most abundant blood vessels. And both doctors take three measurements and take the average vascular index (VI) value by delineating the edge of the lesion on this plane. The entire SMI examination procedure is expected to take less than 5 minutes and the results of the preoperative SMI examination weren’t affecting the patient’s clinical decision.

### Image Evaluation in Training Set and Validation Set

**Figure 3:**
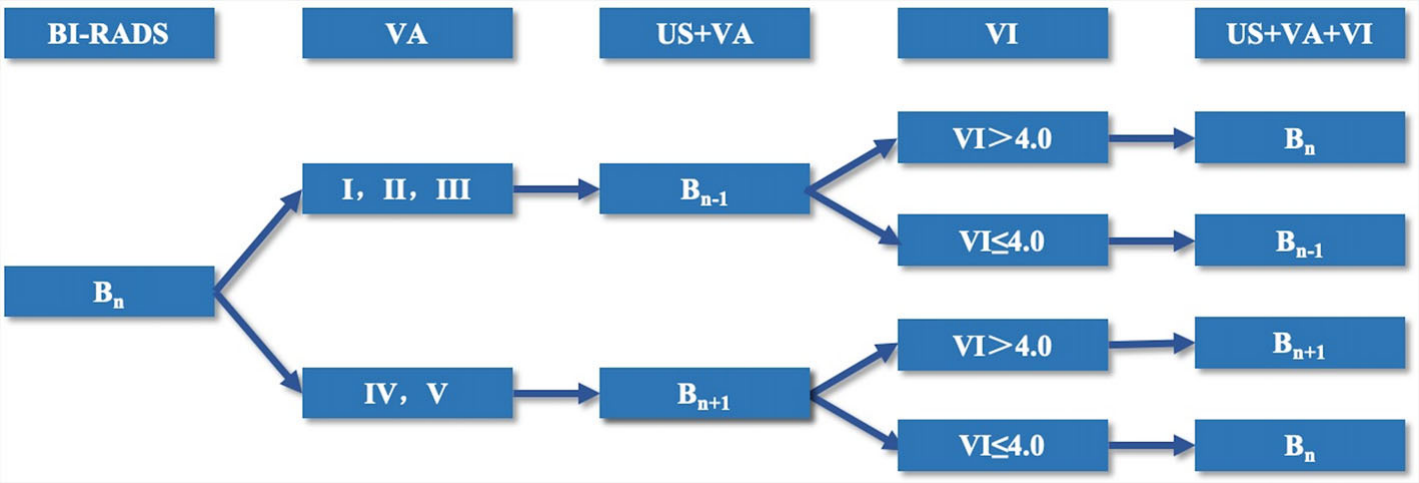
Flow chart of the adjustment mode of category BI-RADS 4 lesions.

In the training set, the benign and malignant cut-off VI values and VA classifications were calculated based on pathological results. Cases in the validation set were graded with BI-RADS after the routine ultrasound examination. Then, the VI cut-off value and VA classifications in the training set were used to upgrade and downgrade the BI-RADS 4 category lesions in the validation set (**Figure 3**). The diagnostic efficiency of conventional ultrasound, conventional ultrasound combined with the VI value, conventional ultrasound combined with the VA, conventional ultrasound combined with the VI value, and the VA were evaluated, and the diagnostic sensitivity, specificity, accuracy and PPV were calculated.

### Statistical Analysis

MedCalc (Windows version 15.2.2; Mariakerke, Belgium) and SPSS (Version 20.0 for Windows; SPSS, Inc., Chicago, IL, USA) were used for statistical analysis. The measurement data were expressed as the mean ± standard deviation (x ± s), and the chi-square test was used for count data. Consistency among observers was assessed by the Weighted Kappa test. Bland-Altman scatter plots were used to analyze the 95% confidence interval between observers of the VI between the two physicians. An index to judge the VI values of benign and malignant breast lesion thresholds was applied, and each VA PPV and NPV were calculated with the receiver operating characteristic (ROC) curve as the reference standard. The DeLong method and others were adopted for the comparison of the ROC curve. A P value less than 0.05 was considered a statistically significant difference.

## Results

### Training Set

#### Pathological Results of Breast Lesions in the Training Set Patients

Among the 225 breast lesions, 112 cases (53.7%) were malignant, and 113 cases (46.3%) were benign (**Table 1**). The mean age of patients in the malignant group (51.3 ± 12.4 years) was higher than that of the benign group (41.0 ± 11.0 years) (P<0.001). The lesion size of patients in the malignant group (2.8 ± 1.6 cm) was larger than that of those in the benign group (1.9 ± 1.3 cm) (P<0.001).

**Table 1:** Histological diagnosis of the lesions confirmed by pathology in training set and validation set.

Training set		Validation set	
Histologic features	N(%)	Histologic features	N(%)
Malignant lesions		Malignant lesions	
Invasive ductal carcinoma	83(36.9)	Invasive ductal carcinoma	110(46.2)
Ductal carcinoma in situ	17(7.6)	Ductal carcinoma in situ	16(6.7)
Solid papillary carcinoma	3(1.3)	Solid papillary carcinoma	5(2.2)
Mucous carcinoma	3(1.3)	Mucous carcinoma	4(1.7)
Invasive lobular carcinoma	2(0.9)	Invasive lobular carcinoma	2(0.8)
Malignant phyllodes tumor	2(0.9)	Malignant phyllodes tumor	0(0.0)
Neuroendocrine carcinoma	1(0.4)	Neuroendocrine carcinoma	0(0.0)
Paget’s disease	1(0.4)	Paget’s disease	0(0.0)
Lobular carcinoma in situ	0(0.0)	Lobular carcinoma in situ	1(0.4)
Total	112(49.8)	Total	138(58.0)
Benign lesions		Benign lesions	
Fibroadenoma	50(22.2)	Fibroadenoma	39(16.4)
Mammary adenosis	29(12.9)	Mammary adenosis	33(13.9)
Intraductal papilloma	23(10.2)	Intraductal papilloma	17(7.1)
Mastitis	5(2.2)	Mastitis	5(2.2)
Benign phyllodes tumor	2(0.9)	Benign phyllodes tumor	3(1.3)
Normal mammary tissue	2(0.9)	Normal mammary tissue	2(0.8)
Mammary cyst	2(0.9)	Mammary cyst	1(0.4)
Total	113(50.2)	Total	100(42.0)
Total	225(100.0)	Total	238(100.0)

#### Consistency Test of the Training Set in Evaluating the VI and VA

Bland-Altman plot analysis showed that the 95% consistency limit of the difference in the VIs measured by the two doctors was -5.55-5.06 (P=0.166), and 4.89% (11/225) were outside the 95% consistency limit. Within the range of consistency, the absolute value of the difference between the VI values measured by the two doctors (a doctor with 15 years’ experience in breast ultrasound examination and a doctor with 3 years’ experience in breast ultrasound examination) was 5.55, the average value of the difference was -0.25, and the value of 95.11% was within the clinically allowable range, showing good consistency. In the weighted kappa analysis of the VAs evaluated by the two doctors (a doctor with 4 years of breast ultrasound experience and a doctor with 3 years of breast ultrasound experience) in 225 cases, the results showed that the VA assessment of 179 cases (79.6%) was consistent between the two doctors. The weighted Kappa coefficient of the VAs of the two sonologists was 0.839 (95% confidence interval of 0.795-0.881, P< 0.001), showing strong inter-observer consistency.

#### Cut-Off Value of the VI in the Training Set of Benign and Malignant Breast Lesions

The ROC curve was used to determine the maximum sensitivity and specificity. The VI value in the malignant group (10.3 ± 8.0) was significantly higher than that in the benign group (4.3 ± 5.0) (P<0.001), and the area under the VI curve was 0.765 (0.704-0.826). The VI value with the maximum value of the Youden index was taken as the threshold value for the diagnosis of benign and malignant lesions. The diagnostic sensitivity was 80.5%, the specificity was 61.9%, the accuracy was 71.1%, the PPV was 62.9%, and the NPV was 76.9% (**Table 2**).

**Table 2:** VI diagnostic performance with different cut-off values.

VI value	Sensitivity	Specificity	Yoden index
3.85	0.805	0.584	0.389
3.95	0.805	0.602	0.407
**4.05**	**0.805**	**0.619**	**0.424**
4.15	0.788	0.628	0.416
4.25	0.770	0.628	0.398

The bold values: the cutoff value and its diagnostic performance.

Among the 225 cases, there were 22 cases (22/112, 19.6%) with malignant lesions with a VI value ≤ 4.0, among which 13 cases (13/22, 59.1%) were invasive ductal carcinoma as the most common pathological type. There were 43 benign lesions (43/113, 38.1%) with a VI value >4.0, among which the most common pathological type was intraductal papilloma in 15 cases (15/43, 34.9%).

#### Smart 3D-SMI VA Typing for Benign and Malignant Breast Lesions in the Training Set

The VA of 225 patients was as follows: 19 cases (8.4%) of type I (PPV 10.5%, NPV 46.6%); 112 cases (49.8%) of type II (PPV 30.4% and NPV 31.0%); 27 cases (12.0%) of type III (PPV 40.7%, NPV 49.0%); 33 cases (14.7%) of type IV (PPV 93.9%, NPV 57.8%); and 34 cases (15.1%) of Type V (PPV 100.0%, NPV 59.2%) (**Table 3**) The VA classification of benign and malignant lesions showed statistically significant differences (P < 0.001). When types IV and V are malignant architecture, and types I, II, and III are benign architecture, the diagnostic sensitivity was 58.0%, the specificity was 98.2%, the accuracy was 78.2%, the PPV was 97.0%, the NPV was 70.3%, and the AUC was 0.781 (95%CI: 0.719-0.844).

**Table 3:** Classification and diagnostic efficacy of 3D-SMI VA in benign and malignant lesions.

	Type I	Type II	Type III	Type IV	Type V	Total
**Malignant (N,%)**	2(0.9)	34(15.1)	11(4.9)	31(13.8)	34(15.1)	112(49.8)
**Benign (N,%)**	17(7.6)	78(34.7)	16(7.1)	2(0.9)	0(0.0)	113(50.2)
**Total (N,%)**	19(8.4)	112(49.8)	27(12.0)	33(14.7)	34(15.1)	225(100.0)
**PPV**	10.5	30.4	40.7	93.9	100.0	
**NPV**	53.4	69.0	51.3	42.2	40.8	
**P value**	0.001	<0.001	0.305	<0.001	<0.001	

Type I, no-vascular pattern; Type II, dotted and linear pattern; Type III, tree-like pattern; Type IV, residual root-like pattern; Type V, crab claw-like pattern.

Among the 225 cases, a total of 48 cases (48/112,42.9%) of malignant lesions were assessed as types I, II or III, among which 32 cases (32/48, 66.7%) were invasive ductal carcinoma as the most common pathological type. A total of 4 cases (4/113, 3.5%) of benign lesions were assessed as types IV and V, among which 2 cases (2/4, 50.0%) were intraductal papilloma as the most common pathological type.

### Validation Set

#### Pathological Results of Breast Lesions in the Validation Set Patients

Among the 238 breast lesions, 138 cases (58.0%) were malignant, and 100 cases (42.0%) were benign (**Table 1**). The mean age of patients in the malignant group (51.7 ± 12.8 years) was higher than that of those in the benign group (43.3 ± 12.3 years) (P<0.001). The mean lesion size in the malignant group (2.5 ± 1.2 cm) was significantly larger than that in the benign group (1.7 ± 1.0 cm) (P<0.001). Compared with the training set, there were no statistically significant differences in patient age, lesion size, VI value or VA type of benign and malignant lesions (**Table 4**).

**Table 4:** The clinical and SMI characteristics of training set and validation set.

	Training set	Validation set	P value
**Age**			
Benign	41.0 ± 11.0	43.3 ± 12.3	0.1661
Malignant	51.3 ± 12.4	51.7 ± 12.8	0.7926
**Size**			
Benign	1.9 ± 1.3	1.7 ± 1.0	0.3334
Malignant	2.8 ± 1.6	2.5 ± 1.2	0.0626
**VI**			
Benign	4.3 ± 5.0	3.8 ± 4.3	0.4399
Malignant	10.3 ± 8.0	9.3 ± 10.4	0.4132
**VA**			0.3590
I	19	18	
II	114	106	
III	27	26	
IV	34	53	
V	35	34	

VI, vasculat index; VA, vascular art architecture; Type I, no vascular pattern; Type II, dotted and linear pattern; Type III, tree-like pattern; Type IV, residual root-like pattern; Type V, crab claw-like pattern.

#### BI-RADS Adjustment Mode of the Validation Set

According to the VI value and VA assessed by SMI, the category of BI-RADS 4 lesions was adjusted. When the Smart 3D-SMI VA was classified as benign types I, II or III, the original BI-RADS category was downgraded by one level. When the VI value of the lesion was greater than 4.0, the original BI-RADS category was upgraded one level to be restored to the original level. When the VI value of the lesion was less than or equal to 4.0, the original BI-RADS category remained at the downgraded level. When the Smart 3D-SMI VA was classified as malignant types IV or V, the original BI-RADS category was upgraded by one level, and when the VI value greater than 4.0, the BI-RADS grading was maintained as upgraded; when the VI value was less than or equal to 4.0, the BI-RADS category was downgraded to maintain the original BI-RADS grade (**Figure 3**). In this study, BI-RADS 3 and BI-RADS 4A categories were selected as the cut-off points for the diagnosis of benign and malignant breast lesions; thus, BI-RADS 3 was set as the benign group, and BI-RADS 4,5were selected as the malignant group to draw ROC curves to analyse the diagnostic efficacy.

#### Diagnostic Efficiency of BI-RADS Classification in the Validation Set and Combined With SMI

**Figure 4:**
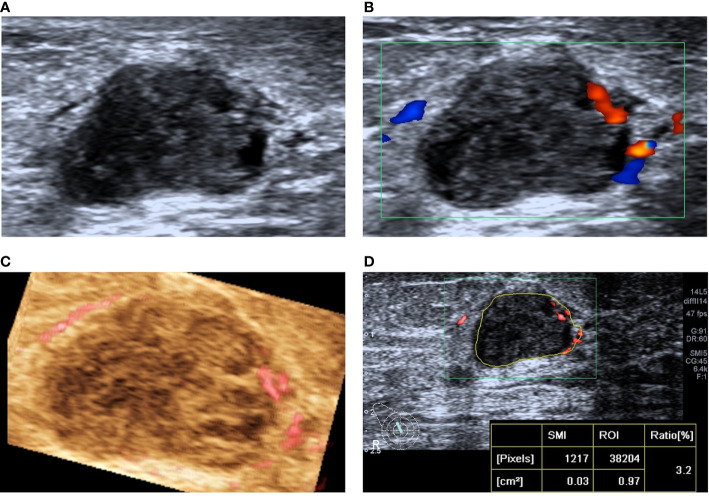
A 78-year-old woman with intraductal papilloma was downgraded from BI-RADS 4A to BI-RADS 3 after SMI assessment. **(A)** Two-dimensional ultrasound shows an irregular hypoechoic lesion. **(B)** CDFI shows the striated blood flow signal around the lesion. **(C)** SMI shows a dotted and linear VA. **(D)** The vascular index was measured by delineating the lesion edge on the plane with the most abundant blood vessels, and the value was 3.2.

**Figure 5:**
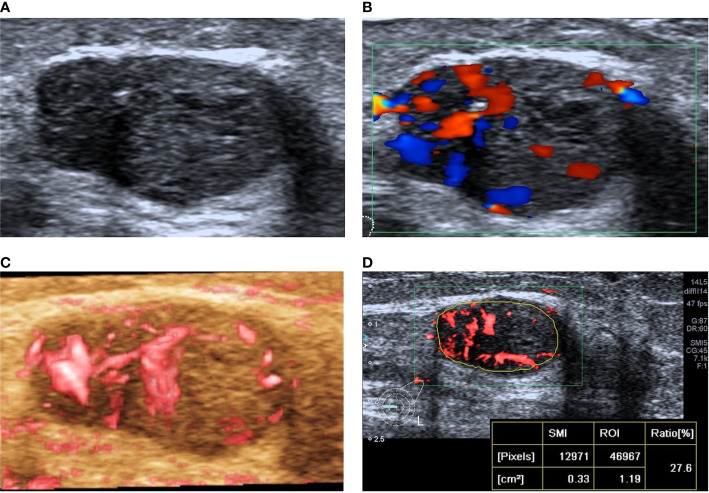
A 31-year-old woman with invasive ductal carcinoma was upgraded from BI-RADS 4C to BI-RADS 5 after SMI assessment. **(A)** Two-dimensional ultrasonography shows an irregular hypoechoic lesion, and punctiform microcalcification was observed. **(B)** CDFI shows abundant blood flow signals around and inside the lesion. **(C)** SMI shows a claw crab-like VA. **(D)** The vascular index was measured by delineating the lesion edge on the plane with the most abundant blood vessels, and the value was 27.6.

When US was combined with the VI, 15 lesions were downgraded, among which 1 malignant lesion was wrongly downgraded; the pathological type was invasive ductal carcinoma, and the nodular size was 0.4 cm. When US was combined with the VA, 23 lesions were downgraded, and 4 malignant lesions were mistakenly degraded, including 2 invasive ductal carcinomas, 1 solid papillary carcinoma, and 1 intraductal carcinoma. The average size of the lesions was 1.2 ± 0.7 cm. When US was combined with the VI and VA, 19 lesions were downgraded, and no erroneous downgraded lesions were found (**Figures 4** and **5**).

**Figure 6:**
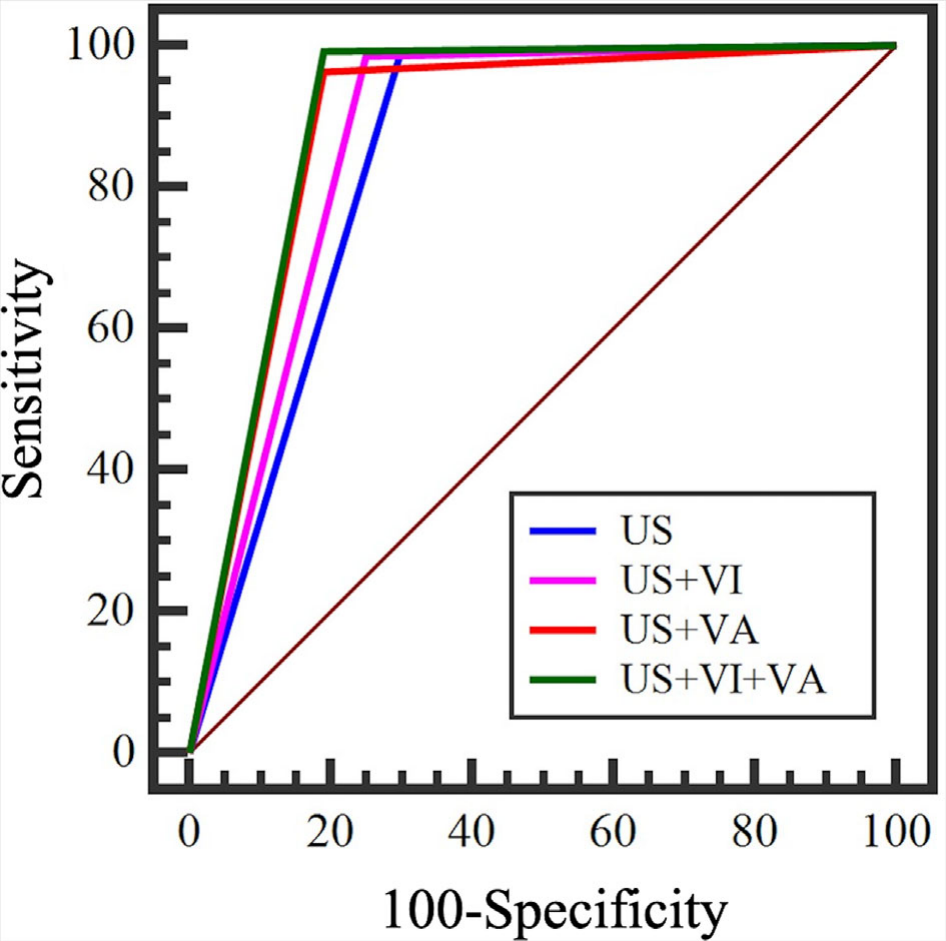
ROC curve of conventional ultrasound combined with SMI.

The ROC curves of US+VI, US+VA and US+VI+VA were significantly different from that of US diagnosis alone. US+VI+VA had the highest diagnostic efficacy, sensitivity (99.3%), specificity (90.0%), accuracy (96.6%), and PPV (93.2%), and the area under the ROC curve was 0.941 (95%CI: 0904-0.979); compared with US diagnosis alone, the difference of the area under the ROC curve was 0.0950, and the Z-statistic was 4.819 (P < 0.001); compared with US +VI, the difference of area under the ROC curve was 0.0286, and the Z-statistic was 2.481 (P=0.0131); compared with US+VA, the difference of the area under the ROC curve was 0.0144, and the Z-statistic was 2.022 (P=0.0432). The difference of the area under the ROC curve between US+VI and US+VA was 0.0142, and the Z-statistic was 1.130 (P=0.2586) (**Table 5**) (**Figure 6**). By comparison of US+VI+VA diagnosis with other diagnostic methods, the difference of the area under the ROC curve was statistically significant, which significantly improved the diagnostic specificity, accuracy and PPV and did not reduce the diagnostic sensitivity.

**Table 5:** Diagnostic efficacy of SMI combined with conventional ultrasound.

	Sensitivity(%)	Specificity	Accuracy性(%)	PPV	AUC	95%CI	P value
**US**	99.3%	71.0%	87.4%	82.5%	0.846	0.789-0.903	0.0002^a^
**US+VI**	98.6%	85.0%	92.9%	90.1%	0.913	0.868-0.957	0.0131^b^
**US+VA**	96.4%	90.0%	93.7%	93.1%	0.927	0.887-0.967	0.0001^c^
**US+VI+VA**	99.3%	90.0%	96.6%	93.2%	0.941	0.904-0.979	0.0432^b^

P^a^, compared with US+VI; P^b^, compared with US +VI+VA; P^c^ compared with US; P^d^ compared with US+VA.

Comparison between US+VI and US+VA, P=0.2586; Compared with US+VI+VA, P < 0.0001.

## Discussion

Tumor growth and metastasis depend on tumor angiogenesis, which involves both the binding of existing host blood vessels to the tumor and the formation of tumor neovascularization (24, 25). The hemodynamic changes accompanied by tumor angiogenesis provide evidence for imaging differentiation of benign and malignant breast lesions. Ultrasound is a commonly used imaging method to detect breast lesions. It is helpful to differentiate benign from malignant diseases and even predict the prognosis of tumors by evaluating the blood vessels (26). SMI is an innovative Doppler ultrasound technology that uses adaptive algorithms to visualize microvessels without contrast. SMI can display microvessel flow signals that cannot be detected by CDFI (27). In our study, the diagnosis of US combined with SMI had the highest diagnostic efficiency with ROC curve was 0.941, which was significantly different from other groups (P<0.05), unnecessary biopsies of benign lesions are reduced.

Smart 3D-SMI automatically calculates the vascular index(VI) based on the delineation of a region of interest (ROI) in the section with the most abundant blood flow. Our results reveal that the mean VI value of malignant breast lesions was significantly higher than that of benign breast lesions, taking pathology as the reference standard, a VI value of 4.05 is a cut-off value with good diagnostic efficacy, which is consistent with the findings of a previous study (20, 28). In our study, the most common pathological type of benign lesions that overlapped with the VI value of malignant lesions was intraductal papilloma, which may be due to the high proportion of tumor cells and stromal cells, which can lead to rich blood flow (20). The most common pathological type of malignant lesions that overlapped with the VI value of benign lesions was infiltrating ductal carcinoma. The angiogenesis of breast cancer is heterogeneous and highly distorted. The rapid growth of the tumor leads to necrosis, degeneration and calcification in the central area of the tumor, which makes it full of connective tissue and fibrous tissue and hard in texture, which is not conducive to angiogenesis (29–31). At the same time, some special pathological types of breast cancers are deficient in blood supply, such as mucinous carcinoma, which is rich in a large amount of mucin, with few stromal components and little angiogenesis (32).

The structure of new blood vessels in breast cancer is different from that in normal tissue (32). Compared with benign lesions, due to tumor angiogenesis regulation factor(such as VEGF and angiogenin) imbalances, highly disordered vascular tissues, such as altered diameter expansion, radial penetration from the edge of the tumor, excessive branch shunts, abnormal structure and function at the tumor edge, radial penetration and other structural and functional abnormalities (32–35). VA analysis is of great significance for obtaining information on the spatial heterogeneity of tumors and differentiating benign from malignant lesions. In our study, there was a significant difference in the VA between benign and malignant lesions, which was consistent with previous research results (10). The residual root-like pattern and crab claw-like pattern are specific signs of malignant breast lesions. Hypoxia and proliferation of tumor cells induce massive neovascularization, leading to vascular remodeling with vascular malformation and arteriovenous fistula formation. The blood flow in the lesion is distorted, and the surrounding area is mostly spinous or radiating vessels (33). The internal blood flow in benign lesions is flat and natural, with peripheral circumferential vessels more common (9). Among them, there was far more false-negative cases than false-positive cases, and most of the pathological types were invasive ductal carcinoma. The reasons for this finding might be as follows: first, the vascular heterogeneity of breast cancer is strong, and the central area of malignant tumors is prone to necrosis, degeneration and calcification, which is not conducive to angiogenesis (36, 37). In this study, only 4 false-positive cases were confirmed in the validation set, and most of the pathological types were intraductal papilloma, which is a hypervascular benign tumor, may partly overlap with the vascular distribution characteristics of malignant lesions. At the same time, when it is accompanied by atypical hyperplasia, the high expression of growth factors such as VEGF allows it to cross-alias with the VA manifestations of malignant lesions to a certain extent (38).

In summary, the SMI technique can quantitatively evaluate the degree of blood flow richness in lesions and show that the vascular structure, VI value and VA of breast lesions, which are related to the benign and malignant breast lesions. Combining conventional ultrasound and SMI technology can improve the diagnostic accuracy, specificity and PPV of BI-RADS and reduce unnecessary biopsies of benign lesions.

Limitations of this study: In this study, no further group discussion was conducted on the relationship between different lesion sizes, different pathological types and VI and VA. Considering the relatively small number of cases after grouping, it may be necessary to further expand the sample size for group discussion.

## Conclusion

A VI value of 4.05 is a cut-off value with good diagnostic efficacy. The hair-like and crab claw-like VA patterns are the characteristic VA patterns of malignant lesions, and the non-vascular, dot-linear and tree-like VA patterns are the characteristic VA patterns of benign lesions. SMI combined with the VI and VA has improved diagnostic efficiency, which can improve the diagnostic specificity, accuracy and PPV without reducing the diagnostic sensitivity.

## Data Availability Statement

The raw data supporting the conclusions of this article will be made available by the authors, without undue reservation.

## Ethics Statement

The studies involving human participants were reviewed and approved by Peking Union Medical College Hospital, Chinese Academy of Medical Science and Peking Union Medical College. The patients/participants provided their written informed consent to participate in this study.

## Author Contributions

Conceptualization: HW. Data curation: SC, XZ, and LZ. Formal analysis: SC and HW. Investigation: HW. Resources: HW, QZ, JL, QS, and YJ. Writing – original draft: SC. All authors contributed to the article and approved the submitted version.

## Funding

This work is supported by Beijing Natural Science Foundation (7202156), Teaching Reform Project of Peking Union Medical College (10023201900113).

## Conflict of Interest

The authors declare that the research was conducted in the absence of any commercial or financial relationships that could be construed as a potential conflict of interest.
